# A cost-effectiveness analysis of mupirocin and chlorhexidine gluconate for *Staphylococcus aureus* decolonization prior to hip and knee arthroplasty in Alberta, Canada compared to standard of care

**DOI:** 10.1186/s13756-019-0568-5

**Published:** 2019-07-11

**Authors:** Elissa Rennert-May, John Conly, Stephanie Smith, Shannon Puloski, Elizabeth Henderson, Flora Au, Braden Manns

**Affiliations:** 1 Departments of Medicine and Community Health Sciences, University of Calgary, Calgary, Canada; 2 Departments of Medicine; Microbiology, Immunology and Infectious Diseases; Pathology and Laboratory Medicine, O’Brien Institute for Public Health; Snyder Institute for Chronic Diseases, University of Calgary, Calgary, Canada; 3 Department of Medicine, University of Alberta, Edmonton, Canada; 4 Department of Surgery, University of Calgary, Calgary, Canada; 5 Department of Community Health Sciences, University of Calgary, Calgary, Canada; 6 Department of Medicine, University of Calgary, Calgary, Canada; 7 Departments of Medicine and Community Health Sciences, O’Brien Institute for Public Health and Libin Cardiovascular Institute, University of Calgary, HRIC Building, 2500 University Drive NW, Calgary, AB T2N1N4 Canada

**Keywords:** Cost-effectiveness analysis, Surgical site infections, Decolonization, Orthopedic surgeries

## Abstract

**Background:**

While decolonization of *Staphylococcus aureus* reduces surgical site infection (SSI) rates following hip and knee arthroplasty, its cost-effectiveness is uncertain. We sought to examine the cost-effectiveness of a decolonization protocol for *Staphylococcus aureus* prior to hip and knee replacement in Alberta compared to standard care – no decolonization.

**Methods:**

Decision analytic models and a probabilistic sensitivity analysis were used for a cost-effectiveness analysis, with the effectiveness of decolonization based on a large published pre- and post- intervention trial. The primary outcomes of the models were infections prevented and health care costs. We modelled the cost-effectiveness of decolonization in a hypothetical cohort of adult patients undergoing hip and knee replacement in Alberta, Canada. Information on the incidence of complex surgical site infections (SSIs), as well as the cost of care for patients with and without SSIs was taken from a provincial infection control database, and health administrative data.

**Results:**

Use of the decolonization bundle was cost saving compared to usual care ($153/person), and resulted in 16 complex *Staphylococcus aureus* SSIs annually as opposed to 32 (with approximately 8000 hip or knee arthroplasties performed). The probabilistic sensitivity analysis demonstrated that the majority (84%) of the time the decolonization bundle was cost saving. The model was robust to one-way sensitivity analyses conducted within plausible ranges. There were small upfront costs associated with using a decolonization protocol, however, this model demonstrated cost savings over one year. In a Markov model that considered the impact of a decolonization bundle over a lifetime as it pertained to the need for subsequent joint replacements and patient quality of life, the bundle still resulted in cost savings ($161/person).

**Conclusions:**

Decolonization for *Staphylococcus aureus* prior to hip and knee replacements resulted in cost savings and fewer SSIs, and should be considered prior to these procedures.

**Electronic supplementary material:**

The online version of this article (10.1186/s13756-019-0568-5) contains supplementary material, which is available to authorized users.

## Introduction

There are over 100,000 hip and knee replacements performed yearly in Canada [1] with approximately 10,000 in Alberta, Canada. While these procedures frequently improve mobility, pain and quality of life, 1–2% of patients develop some level of periprosthetic infection after surgery [2, 3].

This is a severe complication, often requiring multiple hospital admissions, surgeries, and prolonged courses of antibiotics. The Centers for Disease Control and Prevention (CDC) separates surgical site infections (SSI) into superficial incisional, deep incisional and organ space infections [4]. Deep incisional/organ space infections are considered complex and roughly 1% of Albertans undergoing arthroplasty will develop one of these primary infections post-operatively [3].

The Infectious Diseases Society of America (IDSA) guidelines suggest varying management strategies to treat SSI including repeat surgeries and long courses of antibiotics [5]. The exact strategy and type of surgical intervention utilized depend on a number of factors including whether the SSI was complex [5].

The average cost to the Canadian healthcare system per surgical revision of an infected arthroplasty is over $17,000, [6] only considering acute care costs. Even after appropriate surgical and medical management of a primary infection, a proportion of patients (8–60%) will have a relapsed infection and require repeat surgery and potentially chronic antibiotic management [7–11].

Most commonly *Staphylococcus aureus* (*S.aureus*), including the virulent methicillin-resistant *S.aureus* (MRSA), cause post-replacement infections [3, 7, 12]. Though colonization with *S.aureus* is a risk factor for subsequent *S.aureus* SSI, there are no uniform guidelines around decolonization pre-operatively.

In some decolonization protocols chlorhexidine gluconate (CHG) is used as a once daily full body wash for up to 5 days at the same time as using intranasal mupirocin antibiotic ointment to the nares twice a day. For optimal efficiency, this protocol should be initiated within 10–14 days (but no more than 30 days before an operation). Usually the decolonization protocol is used for approximately 5 days leading up to the day of surgery [13].

A prior study completed in the US in 2012 examined the cost utility of nasal mupirocin for *S.aureus* decolonization prior to hip and knee arthroplasty. This study used a simple tree model to look at a “screen for *S.aureus* and decolonize” versus “universal decolonization protocol” versus “standard of care – no decolonization” [14]. The results suggested that a “screen and treat” and a “treat all” method were cost-effective compared to no treatment. However, the authors suggested that treating everyone is more practical and should be the strategy of choice [14].

We sought to evaluate if an evidence based *S.aureus* decolonization protocol, including intranasal mupirocin and CHG, implemented in all adult patients prior to knee and hip replacement in Alberta, compared with standard care (no routine decolonization) was cost-effective.

## Methods

### Economic evaluation

A cost effectiveness analysis (CEA) was conducted to assess the efficiency of implementing a decolonization protocol prior to hip and knee arthroplasty in Alberta. The analysis assessed the impact on costs and the number of infections avoided.

### Study population

The target population for this analysis was all adult patients (> 18 years of age) in Alberta who receive elective knee or hip arthroplasty. Data for this cohort was based on 24,667 patients who underwent primary elective hip or knee arthroplasty in Alberta between April 1, 2012 and March 31, 2015 (mean age 66.5 years) [15]. No subgroups were analyzed in this study as all adults undergoing joint replacement may derive benefit from decolonization.

### Study setting

In Alberta, nearly all patients who receive an elective hip and knee arthroplasty are triaged and managed through centralized hip and knee clinics and are assigned a nurse case manager. These clinics are the medical facilities where information and decolonization materials could be supplied to the patients. Patients receive both pre-operative and post-operative follow up care at these clinics.

### Perspective

The analysis was done from the perspective of the publicly funded health care system, and as such, included the costs of the inpatient hospitalizations and outpatient ambulatory care visits.

### Effectiveness

The effectiveness of a *S.aureus* decolonization protocol at reducing *S.aureus* complex SSI was derived from a pre- and post-intervention trial [13]. The pre- and post-intervention study examined 31 701 hip and knee arthroplasties (20642 pre-intervention and 11059 post intervention) completed at 16 different hospitals in the US. In the pre-intervention phase patients were treated with routine pre-operative antibiotics. In the post intervention phase patients were screened for *S.aureus* nasal carriage and if positive, were treated with 5 days of CHG (2% cloths or 4% body wash) baths and twice daily 2% intranasal mupirocin. They then received appropriate pre-operative antibiotics. This intervention was administered pre-operatively in both the pre- and post-intervention groups. The outcome measured was the number of complex SSIs caused by *S.aureus.* The rate ratio of complex *S. aureus* SSIs for hip and knee arthroplasties in the post-intervention group compared to the pre-intervention group was 0.51 [13].

A factor that influenced effectiveness of the model was compliance which was taken into account in the pre- and post-intervention study, where only 83% of patients were adherent to the decolonization protocol (39% fully adherent and 44% partially adherent). Another factor that influenced effectiveness was the number of patients with a complex *S.aureus* SSI. If there were very few *S.aureus* infections then it was anticipated that there would be a loss of effectiveness.

### Time horizon

In the baseline analysis, the time horizon used was 1 year, as most SSIs following knee and hip arthroplasty occur within the first 3 months post-operatively with a large proportion occurring within the first 30 days [3].

### Statistical analyses

#### Base case model and data considerations

**Fig. 1:**
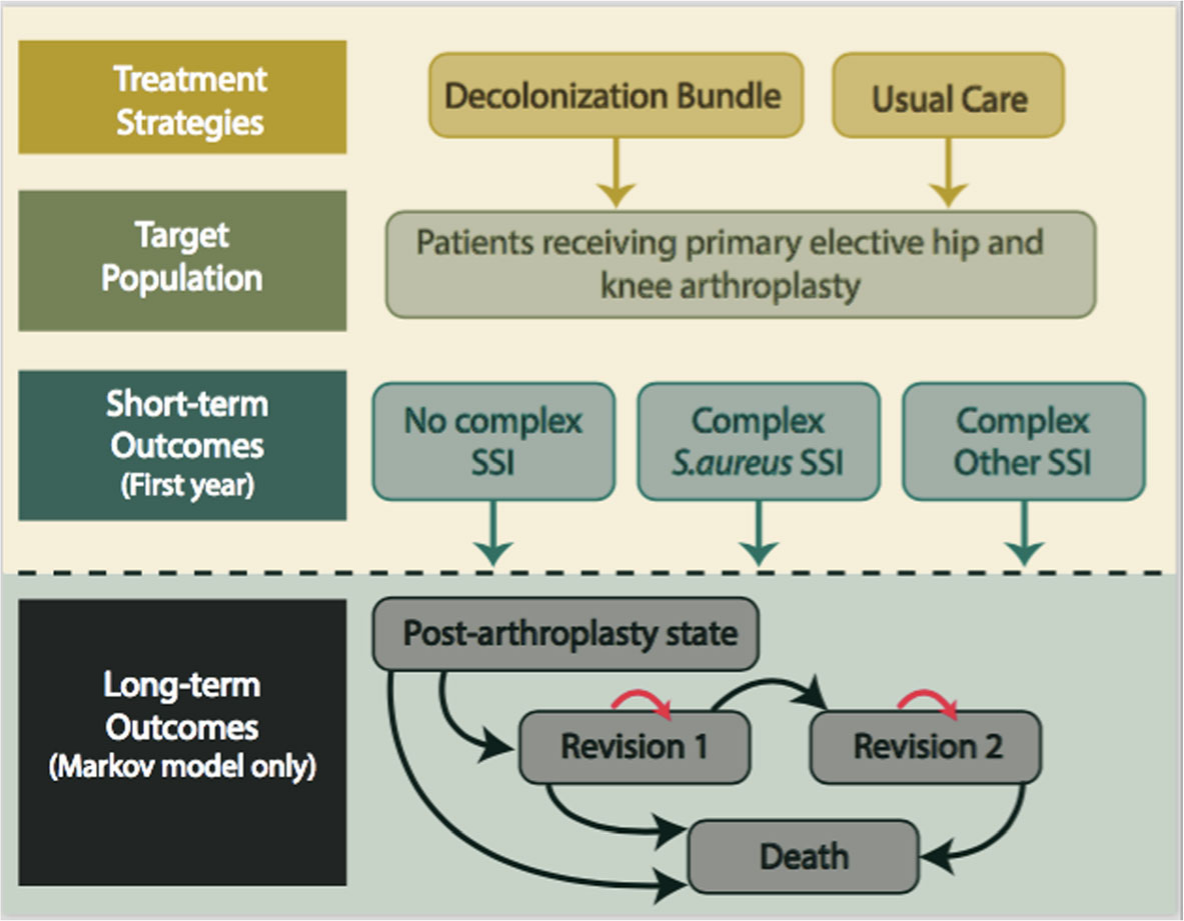
Simple tree and Markov model for patients undergoing total hip or knee arthroplasty who either have no decolonization treatment or receive mupirocin and CHG decolonization for *S.aureus* prior to surgery. Abbreviations: *S.aureus* = *Staphylococcus aureus,* SSI = surgical site infection

The model used was a simple decision tree (Fig. 1) and was constructed using decision analysis software (TreeAge Pro 2018 Williamstown, MA). The model was evaluated by clinical experts in order to establish face validity. A wide range of sensitivity analyses were undertaken to ensure the model responded appropriately.

The incidence and causative pathogens of complex SSI occurring in all adults in Alberta following knee and hip replacement from April 1 2012 to March 31 2015 were collected from a previous study that we completed on this population [15]. Data inputs are displayed in Table 1.

**Table 1:** Model inputs by treatment alternatives

Variable	Usual Care (No decolonization)	Decolonization Bundle	Ranges for Inputs (95% CI unless otherwise stated)	Source	Distribution for PSA
Risk of *S.aureus* complex SSI	0.40%		0.338–0.464	AHS IPC Database	Beta
Risk of other complex SSI	0.64%	0.64%	0.576–0.703	AHS IPC Database	Beta
RR for *S.aureus* SSI with decolonization bundle		0.51	0.3–0.85	Schweizer et al. [13]	Beta
Mupirocin Cost/ person ($)^a^	0	2.30	1.15–3.45	Alberta Blue Cross Formulary	Gamma
Chlorhexidine Cost/person ($)^a^	0	4.95	2.47–7.43	Dufort Lavigne Website [16]	Gamma
Cost of Nurse Educator/ person ($)^a^	0	13.35	6.68–20.03	AHS Job Posting for Infection Control Professional	Gamma
1 year costs SSI with *S. aureus*($)	108,175	108,175	88,223-128,127	Rennert-May et al. [15]	Gamma
1 year costs SSI with other pathogen($)	87,317	87,317	79,830-94,804	Rennert-May et al. [15]	Gamma
1 year costs with no infection ($)	19,893	19,893	11,216-28,570	Rennert-May et al. [15]	Gamma

*Abbreviations*: *SSI* Surgical Site Infection, *AHS* Alberta Health Services, *IPC* Infection Prevention and Control, *S.aureus Staphylococcus aureus, PSA* Probabilistic sensitivity analysis, *RR* rate ratio

^a^Ranges for cost created by adding and subtracting 50%

#### Base case cost inputs

The cost for the mupirocin ointment was taken from the Alberta Blue Cross Formulary. The cost per gram was 45 cents and a 5-g tube would be issued. The cost of 4% CHG sponges was taken from a medical supply website [16] and five would be needed for each patient. It was assumed that in order to implement the decolonization protocol at all hip and knee clinics across Alberta, a nurse educator in Infection Prevention and Control would need to be hired to educate medical staff and create instructional handouts for patients. This cost was estimated based on AHS job postings for nurse educators. When taking into account the number of patients receiving hip and knee replacement annually in Alberta this cost $13.35 per person (including an additional 21% increase on the hourly salary to account for benefits). The total cost for all components of the decolonization bundle was $20.60 per person.

Costs for hospitalizations and management of SSIs as well as the cost of the initial arthroplasty were obtained from a population based cohort study including all patients in Alberta undergoing hip and knee arthroplasty [15]. The costs were a combination of average costs and microcosting data yielding very high quality costing estimates [15]. These costs encompassed inpatient hospitalizations and outpatient visits but did not include patient borne costs (e.g. travel, outpatient antibiotics) or additional physician remuneration.

No discount rate was applied as the time horizon was 1 year. All costs were inflated to 2016 CDN$. Costs (and infections avoided) were the output considered in the model. Utilities, life years and quality adjusted life years were not considered in this base model given the short time horizon. Costing inputs are represented in Table 1.

#### Scenario and sensitivity analyses

A one-way sensitivity analysis was completed to estimate the influence that the range of input values had on the overall costs associated with using a bundle. Each input variable was varied one at a time using the 95% confidence intervals. For bundle component costs, dollar values were varied in each direction by 50%. A probabilistic sensitivity analysis (PSA) where we allowed for all variables to change simultaneously (though 1000 Monte Carlo simulations) was completed using distributional assumptions of the input parameters (Table 1).

We considered a scenario where no nurse educator was required as eventually the decolonization bundle could be implemented as part of the normal hip and knee clinic protocol. Additionally, a scenario was conducted where compliance was reduced by 50%. For the purposes of this scenario, we assumed that a reduction in compliance of 50% would increase the rate ratio of *S.aureus* by 50%, i.e. 0.77.

Finally, to assess model uncertainty, we developed a Markov model (Fig. 1) to examine the long-term impacts of developing a SSI by modeling revision arthroplasty over a lifetime in those who had received a decolonization protocol versus those who did not. It is generally accepted that every time a revision procedure is performed there is an increased risk of requiring another surgical intervention [17]. As almost all patients with a complex SSI will receive some form of surgical procedure in conjunction with the IDSA guidelines [5], these patients would be at a greater risk of requiring further surgical interventions compared to those who only received an initial arthroplasty. As such, this long-term model enables consideration of the impact of repeated surgeries and having a complex SSI on the need for joint replacements in the future, and the utility of existing in each of these long-term states. This allows for a more complete assessment of how uncertainty in effectiveness impacts results. Further details regarding this model can be found in Additional file 1.

#### Model validity

This model was validated using guidelines for economic evaluations [18]. Content experts were involved throughout the model creation to ensure face validity. Statistical methods that had been used previously for determining cost inputs were validated [15]. The coding accuracy of the model was tested by changing values to extremes and ensuring the model appropriately responded.

## Results

### Base case

**Fig. 2:**
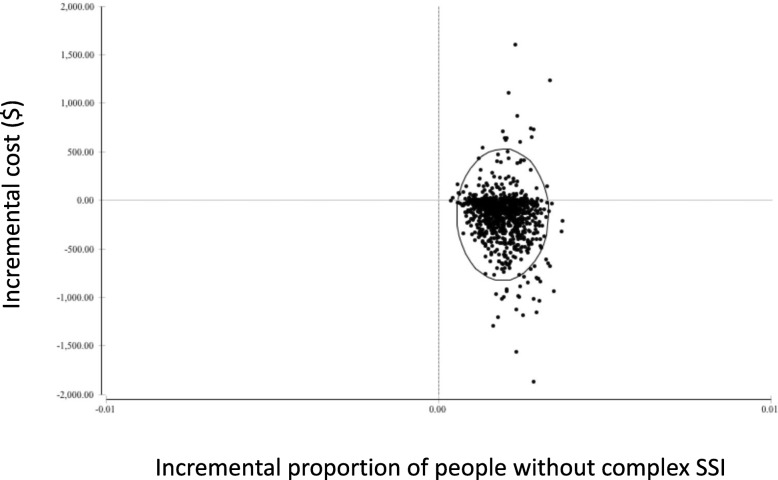
Scatterplot of the probabilistic sensitivity analysis demonstrating that with 1000 iterations, 84% of the time the decolonization protocol is less costly and more effective. Quadrant I is more effective and more costly, quadrant II is more effective and less costly, quadrant III is less effective and less costly, and quadrant IV is less effective and more costly

In our base case analysis, the average cost for those who received the decolonization protocol was $20,525 and for those who received standard of care the cost was $20,678 (a cost savings of approximately $153 per person, which in Alberta translates into savings of $1.26 million annually). The risk of developing a *S.aureus* complex SSI was reduced from 0.4 to 0.2%|. This change in effectiveness translates into 16 complex SSIs from *S.aureus* annually in Alberta when a decolonization bundle is used versus 32 complex SSIs with standard of care. When the PSA was completed with 1000 iterations, 84% of the time it was more effective and less expensive to utilize the decolonization bundle (Fig. 2).

### Sensitivity and scenario analyses

**Fig. 3:**
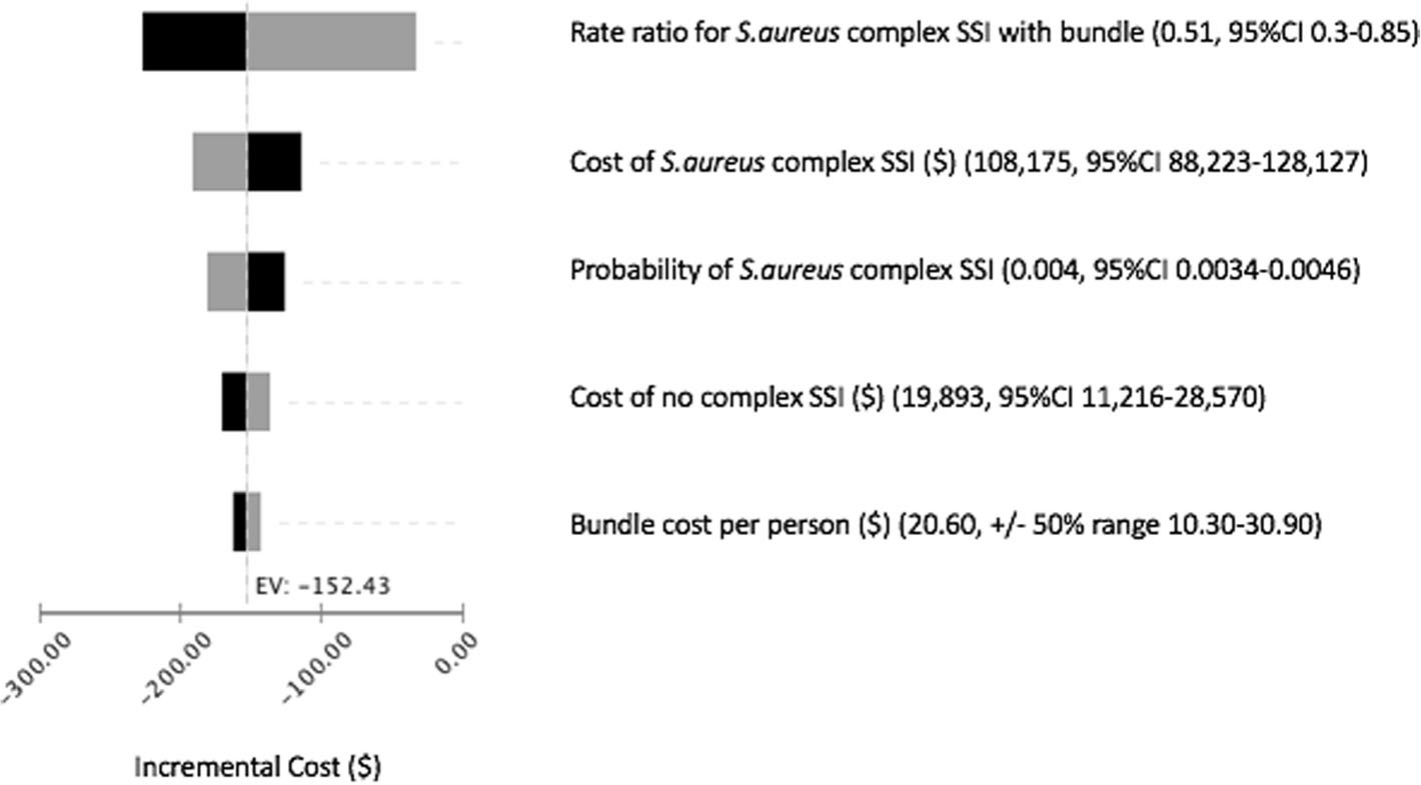
Tornado diagram of one-way sensitivity analyses on the cost difference between treating patients with a decolonization bundle versus standard of care, with a black bar representing the lower end of the range and a grey bar representing the high end of the range. Abbreviations: *S.aureus* = *Staphylococcus aureus,* SSI = surgical site infection

The one-way sensitivity analysis of each estimate completed, demonstrated that the use of the decolonization bundle would result in a cost savings. The smallest impact on the difference in cost between decolonization and no decolonization occurred when the probability of *S.aureus* complex SSI after decolonization, compared to no decolonization, increased. At the upper limit of this 95% confidence interval (when the rate ratio was 0.85), the cost savings for decolonization was only $32.00 per person. The largest impact on the cost difference between decolonization and no decolonization occurred when the rate ratio of *S.aureus* complex SSI was at the lower limit of the range (i.e. 0.3) (cost savings were $227 per person). A tornado diagram that represents only variables that created a difference in incremental costs is provided in Fig. 3.

In the scenario analysis where no nurse educator was required, the savings were $166 per person. In the scenario analysis where compliance was reduced by 50% (hence the rate ratio of *S.aureus* was 0.77), the cost savings were $61 per person.

### Markov model

The Markov model demonstrated that over a lifetime the cost savings for those who were decolonized versus those who were not were $161/person. And the difference in effectiveness (i.e. QALYs) was 0.00096 between those who were decolonized versus those who were not. Additional File 1 provides further detail.

## Discussion

We found the use of the decolonization bundle to prevent *S.aureus* complex SSI resulted in cost savings of $153 per person and 16 SSIs avoided annually (with approximately 8000 hip or knee arthroplasties done). Both the one-way sensitivity analysis and the PSA demonstrated that use of the decolonization bundle generally resulted in fewer complex SSIs, and cost savings, which was consistent with our base model findings. The Markov model discussed further in additional file 1 demonstrated that over a lifetime there were still cost savings for those who received the decolonization bundle – even if complex *S.aureus* SSIs were reduced from only 0.4 to 0.2%.

Our base case findings of reduced SSIs and costs associated with a decolonization protocol are consistent with prior studies in addition to the one mentioned previously [14]. One study created an economic model to examine the cost-effectiveness of universal *S.aureus* decolonization with nasal mupirocin prior to hip and knee arthroplasty and determined that there were associated cost savings and reduced infections [19]. Another recent study examined cost-effectiveness of a decolonization protocol for orthopedic procedures [20]. The authors used effectiveness data for decolonization from a randomized controlled trial they had previously completed, examining the eradication of *S.aureus*. For the cost-effectiveness analysis, they estimated SSI risk from the literature [20]. They found a “treat all” decolonization strategy resulted in the most cost-savings compared to “screen and treat” and “no treatment”. However, their costs were based on average estimates from previous studies and were not specific to the patient population they were studying [20].

Our model was robust to sensitivity analyses demonstrating cost-savings within plausible ranges of the input variables which is consistent with previous studies. In one of the studies mentioned above, their sensitivity analysis demonstrated that the unlikely scenario of infections being reduced by less than 10% and infection related costs being less than $70,000 had to be achieved in order for a decolonization protocol to no longer be cost-saving [19]. In the other study mentioned above [20] they completed both one way sensitivity analyses and a probabilistic sensitivity analysis similar to the current study. In a one-way sensitivity analysis, they noted that a “treat all” approach to decolonization was always more effective and less costly than standard of care. In probabilistic sensitivity analysis, they demonstrated that it was always more effective and less costly to “treat all” with decolonization [20]. Another study demonstrated in their sensitivity analyses that even with modest compliance, it was still cost-saving to utilize home-based CHG bathing cloths pre-operatively to prevent SSIs in patients undergoing orthopedic procedures [21]. This was based on prior studies demonstrating the effectiveness of just CHG pre-operative bathing in reducing SSIs [22, 23]. It should be noted however, in the Schweizer et al. study used for effectiveness in our model [13], when compliance was broken down into full adherence and partial adherence, the partially adherent group did not have a statistically significant reduction in complex SSIs (RR 0.8 95%CI 0 .49–1.31) suggesting a potential need for both mupirocin and CHG bathing. Full adherence for those with *S.aureus* positive screening was defined as any CHG bathing and at least 3 days of mupirocin. Partial adherence was defined as any CHG bathing and any mupirocin use.

There is a risk of *S.aureus* resistance to topical antibiotics that would decrease the effectiveness of a decolonization protocol. In the Schweizer et al. trial, they did test for mupirocin and CHG resistance in 36 *S.aureus* samples and found only one sample with mupirocin resistance [13]. Any increase in resistance would have been reflected in the probability of *S.aureus* with decolonization increasing, which was taken into account in the sensitivity analysis.

Our study uniquely used high quality patient-specific microcosting data and local data regarding volume of complex SSIs and causative pathogens, on a large patient population (*N* = 24,667). We were able to use precise and accurate costing data in our model rather than general estimates from the literature which to our knowledge has not previously been done. While the trial we utilized for effectiveness was not a randomized controlled trial, it was a pragmatic trial with a substantial volume of patients improving the accuracy of the findings. Our model demonstrated that even with a modest reduction in number of infections, decolonization was cost saving. Our study also extended a Markov model of cost-effectiveness of decolonization, specifically in orthopedic surgeries, over a lifetime. This adds value, as while our study was not the first to demonstrate cost-savings with decolonization prior to hip and knee replacements, it considered the long-term impact of CHG and mupirocin decolonization on patient quality of life as well as cost savings. This study also contributes to a paucity of Canadian literature on the cost-effectiveness of decolonization strategies for *S.aureus.* The epidemiology of knee and hip arthroplasty SSIs and costs associated with them are similar across Canada rendering this model relevant and generalizable nationally. While costs in different countries are not identical to Canada, the principles of the model still apply and so it provides valuable information that can be applied globally.

There were several limitations to our model. The trial used to determine effectiveness of decolonization was not a randomized controlled trial. However, even when the rate ratio for developing a complex *S.aureus* SSI after a decolonization bundle was as high as 0.94, in the Markov analysis, cost savings were still noted. Additionally, in this study they did not provide results separately for knee and infections. This should not have impacted the results as risk of infection and response to decolonization is likely very similar in hips and knees. This study also implemented a screen and treat strategy and we extrapolated that the rate ratio would still be applicable to our model with a treat all strategy. This was validated with a previous systematic review and meta-analysis that demonstrated that there was no difference in the effectiveness of decolonization strategies when treat all or screen and treat approaches were used [24].

This trial noted that screening allowed for targeting pre-operative antibiotics more effectively (i.e. vancomycin for MRSA). Targeted prophylaxis was unlikely to make a substantial difference in our outcomes and model which assumed universal decolonization, as rates of MRSA in Canada in hospital patients are fairly low (4%) [25], and pre-operative antibiotics are targeted to cover MRSA when known to be colonized or when there are MRSA risk factors. Finally, unlike our other model inputs that were taken from local data (i.e. costs and rates of complex SSIs) the effectiveness of decolonization was extrapolated from a study conducted in an American setting [13]. However, we feel the study setting was still applicable to our population given that the American study used data from hospitals described as urban facilities, minor teaching centers and community sites ranging from 52 to 514 beds, each having the infrastructure for quality improvement initiatives.

## Conclusions

We determined that a decolonization protocol prior to hip and knee arthroplasty could result in fewer complex *S. aureus* SSIs and cost savings at both 1 year and over a lifetime. Implementation of this bundle would have small effects on health services, requiring only a small outlay for decolonization supplies and a nurse educator. Once the program has been implemented, a dedicated nurse educator would likely no longer be required and the cost savings would increase as demonstrated in our alternate scenario. Since lower extremity arthroplasty procedures are increasing over time, we expect higher savings over time as more infections are prevented, and that this may result in a small additional capacity in operating rooms available for other needed procedures. Given our findings, we believe health systems should implement a decolonization protocol prior to hip and knee arthroplasty and continue surveillance of complex SSIs to determine if there is a reduction as anticipated. Additionally, monitoring for the development of resistance to topical antimicrobials should be pursued, particularly once a decolonization protocol is in place.

## Additional file

Additional file 1: A Markov model assessing the impact of a decolonization bundle for *Staphylococcus aureus* prior to hip and knee arthroplasty, on costs and quality of life over a lifetime. (DOCX 30 kb)

## Data Availability

Data sharing is not applicable to this article as no datasets were generated during the current study.

## Abbreviations

CDC: Centers for Disease Control and Prevention
CEA: Cost-effectiveness analysis
CHG: Chlorhexidine gluconate
IDSA: Infectious Diseases Society of America
QALY: Quality adjusted life year
RR: Rate ratio
*S. aureus*: *Staphylococcus aureus*
SSI: Surgical Site Infections
US: United States
