# Supplementary material for: A cost-effectiveness analysis of mupirocin and chlorhexidine gluconate for Staphylococcus aureus decolonization prior to hip and knee arthroplasty in Alberta, Canada compared to standard of care
Source: Antimicrob Resist Infect Control. 2019 Jul 11;8:113. doi: 10.1186/s13756-019-0568-5 (PMC6625116; doi:10.1186/s13756-019-0568-5)
Supplement: Supplementary file 1 — A Markov model assessing the impact of a decolonization bundle for Staphylococcus aureus prior to hip and knee arthroplasty, on costs and quality of life over a lifetime. (DOCX 30 kb) [file 13756_2019_568_MOESM1_ESM.docx]

**Appendix 1.**

**Markov model**

*Model design and structure*

A Markov model (see Figure 1 from the main text) was created to assess model uncertainty, and to determine the impact of decolonization over a patient’s lifetime on long-term costs and patient outcomes, including the need for subsequent joint replacements (which is increased in patients with complex surgical site infections (SSIs)), and quality of life. It also offered an opportunity to further explore the impact of uncertainty in clinical effectiveness, relevant as the effectiveness of the decolonization bundle was based on a large quasi-experimental trial rather than a randomized controlled trial [1].

The Markov model was designed to determine whether the costs of implementing the decolonization bundle, even if much less effective, could still offset short-term and long-terms costs associated with complex SSIs, which can increase the risk of needing joint replacements in the long-term. This model allowed us to assess the impact of complex SSIs on the increased risk of joint replacements, which we could not take into account in the baseline model. In this model, we assumed that all patients who had a complex SSI would have some sort of surgical management in the following year. By virtue of having an additional surgery, they would be at increased risk of having repeated revision arthroplasties compared to those patients who did not develop a complex SSI, as anytime the joint is revised there is a loss of bone and structure increasing the risk of joint failure [2]. There was no conditional modelling for recurrent SSIs (i.e. we did not consider that patients who had a SSI would be at increased risk of recurrent SSI after subsequent surgery).

The Markov model included four health states. After the initial arthroplasty (in those without SSIs) and subsequent joint revision (in those with SSIs), all patients entered a post-arthroplasty state. Patients could remain in that state (the majority of patients would remain here), require a revision arthroplasty for joint failure (more commonly required in those who developed an SSI due to their increased risk from having a prior additional surgical procedure) and go to health state Revision 1, or die. Following this, patients in Revision state 1 could require an additional revision arthroplasty for another joint failure of the same joint and go to health state Revision 2, or die. The outcomes were quality adjusted life years (QALYs) gained, costs and cost per QALY gained. A QALY combines patient survival with a measure of overall quality of life (utility). They are calculated by multiplying life years by a utility which ranges from 0-1, with 0 being equivalent to death and 1 being synonymous with perfect health. The model was conducted in TreeAge Pro (TreeAge Software 2018 Williamstown, MA) with transitions occurring yearly.

*Model inputs*

The risk of recurrent arthroplasty was taken from a JAMA study on hip arthroplasty [2]. The yearly revision risks per year were 0.01, 0.04 and 0.05 after initial arthroplasty, one revision and two revisions, respectively. At baseline we assumed the annual risk of requiring a revision for those who did and did not develop a complex SSI post arthroplasty was 0.04, and 0.01 respectively. To estimate QALYs, we used utilities from a study on patients undergoing hip arthroplasty [3]. The study provided utilities for patients experiencing an SSI post arthroplasty (0.4) and then what they would increase to when the SSI was cured (0.823) as well as those without an SSI post arthroplasty (0.858) [3]. For patients with an SSI, in this model, we assumed a utility score of 0.4 for the first year, with an increase to 0.823 after year 1. The utilities vary by time and health state (Table 1).

For costs, we utilized data from a cohort study we previously conducted in Alberta of 24,667 patients undergoing hip and knee arthroplasty [4]. The cost of a decolonization bundle was described in the main body of the manuscript. Table 2 demonstrates all other costs and associated explanations for their changes over time. All costs were inflated to 2016 $CDN. Annual mortality rates for the first two years were taken from a previous study conducted on this patient population [4]. For all future years Statistics Canada mortality tables were utilized to calculate the annual risk of mortality [5].

This model was conducted over a lifetime horizon and in accordance with guidelines for economic evaluations a 1.5 percent discount rate was applied [6].

*Sensitivity Analysis*

A one-way sensitivity analysis was conducted varying the rate ratio of *Staphylococcus aureus* (*S.aureus*) complex SSIs to model how ineffective a decolonization bundle might be before it would no longer result in cost savings over a lifetime.

*Model Validity*

This model was assessed by content experts in order to determine face validity. Any methods used to determine costs had previously been evaluated and validated [4].

**Results**

*Base case*

Over a lifetime the cost savings for those who received decolonization were $161/person. QALYs for patients who had received decolonization were 10.48154 and for those who did not receive decolonization they were 10.48058, resulting in a difference of 0.00096. This very small difference was expected given that only 1.04% of patients develop a complex SSI post hip or knee arthroplasty [4] and the decolonization bundle reduces *S.aureus* complex SSIs from 0.4% to 0.2%. The impact of a complex SSI on costs and utilities would predominantly be apparent in the first year post arthroplasty. As we assumed only slightly lower long-term quality of life for those after SSI and after recurrent joint arthroplasty, it is likely an underestimate of the true cost-effectiveness over a lifetime, between those who received decolonization and those who did not.

*Sensitivity analysis*

In order for the Markov model to demonstrate that the decolonization bundle was no longer effective at creating cost savings, the rate ratio for *S.aureus* complex SSIs had to be increased to 0.95. At a rate ratio of 0.94 there were still small cost savings ($2) over a lifetime. Therefore, even if the decolonization bundle was minimally effective and only reduced complex *S.aureus* SSI from 0.4% to 0.38% there would still be cost savings over a patient lifetime.

**References**

1. Schweizer ML, Chiang H-Y, Septimus E, Moody J, Braun B, Hafner J, et al. Association of a bundled intervention with surgical site infections among patients undergoing cardiac, hip, or knee surgery. *JAMA* 2015;313:2162-71.

2. Chang RW, Pellisier JF, Hazen GB. A cost-effectiveness analysis of total hip arthroplasty for osteoarthritis of the hip. *JAMA* 1996;275:858-65.

3. Merollini KM, Crawford RW, Whitehouse SL, Graves N. Surgical site infection prevention following total hip arthroplasty in Australia: a cost-effectiveness analysis. *Am J Infect Control* 2013;41:803-9.

4. Rennert-May ED, Conly J, Smith S, Puloski S, Henderson E, Au F, et al. The cost of managing complex surgical site infections following primary hip and knee arthroplasty: A population-based cohort study in Alberta, Canada. *Infect Control Hosp Epidemiol* 2018;39:1183-88.

5. Statistics Canada. Life tables, Canada, provinces and territories. https://www150.statcan.gc.ca/n1/en/catalogue/84-537-X. 2018. Accessed January 8, 2019.

6. Canadian Agency for Drugs and Technologies in Health.. Guidelines for the Economic Evaluation of Health Technologies: Canada. <https://www.cadth.ca/about-cadth/how-we-do-it/methods-and-guidelines/guidelines-for-the-economic-evaluation-of-health-technologies-canada>. 2017. Accessed April 8, 2019.

7. Rasanen P, Paavolainen P, Sintonen H, Koivisto A, Blom M, Ryynanen O, et al. Effectiveness of hip or knee replacement surgery in terms of quality-adjusted life years and costs. *Acta Ortho* 2007;78:108-15.

**Tables**

Table 1. Model Inputs for utilities with justification

|  | **Parameters for patients with no SSI** | | | **Parameters for patients with SSI** | | |
| --- | --- | --- | --- | --- | --- | --- |
| **Post-arthroplasty state** |  | **Justification** | **Source** |  | **Justification** | **Source** |
| Utility – year 1 | 0.858 | Based on prior study | Merollini et al.^3^ | 0.4 | Based on prior study | Merollini et al.^3^ |
| Utility – years 2 and on | 0.858 | Based on prior study | Merollini et al.^3^ | 0.823 | Based on prior study | Merollini et al.^3^ |
| **Revision arthroplasty state** |  | **Justification** | **Source** |  | **Justification** | **Source** |
| Utility – after revision arthroplasty | 0.81 | Decreased utility value to reflect the need for a revision arthroplasty (utility reverts back to utility pre-initial arthroplasty) | Rasanen et al.^7^ | 0.775 | Decreased utility by same decrement as for those with no SSI who went from post-arthroplasty state to revision surgery | Expert opinion |
| Utility – after repeat revision arthroplasty | 0.762 | Decreased by same decrement to account for requiring additional revision | Expert opinion | 0.727 | Decreased by same decrement to account for requiring additional  revision | Expert opinion |

Abbreviations: SSI=Surgical site infection

Table 2. Model Inputs for costs with justification

|  | **Parameters for patients with no SSI** | | | **Parameters for patients with SSI** | | |
| --- | --- | --- | --- | --- | --- | --- |
| **Post arthroplasty state** |  | **Justification** | **Source** |  | **Justification** | **Source** |
| Cost – year 1 ($) | 19,893 | Based on prior study | Rennert-May et al.^4^ | 87,317 other pathogen  108,175 *S.aureus* | Based on prior study | Rennert-May et al.^4^ |
| Cost – year 2 ($) | 5,250 | Based on prior study for costs the second year after arthroplasty | Rennert-May et al.^4^ | 11,040 | Based on prior study for costs the second year after arthroplasty for those who developed a complex SSI | Rennert-May et al.^4^ |
| Cost – years 3 and on ($) | 5,250 | Based on prior study for costs the second year after arthroplasty | Rennert-May et al.^4^ | 5,250 | The costs equalize between those who developed SSI and those who did not, so the cost is now the same as the non-infected group | Expert opinion |
| **Revision arthroplasty states** |  | **Justification** | **Source** |  | **Justification** | **Source** |
| Cost – additional transient cost for the first year in a revision state (would be added for a one year period to yearly costs described above) | 15,547 | Year 1 costs averaged between those who did and did not have an infection (this would encompass the cost of an arthroplasty and associated care) | Rennert-May et al.^4^ and expert opinion | 15,547 | Year 1 costs averaged between those who did and did not have an infection (this would encompass the cost of an arthroplasty and associated care) | Rennert-May et al.^4^ and expert opinion |
|  |  |  |  |  |  |  |

Abbreviations: SSI=Surgical site infection, *S.aureus*=*Staphylococcus aureus*
